# Supplementary material for: Antimicrobial Resistance Profiles and Genes of Staphylococci Isolated from Mastitic Cow’s Milk in Kenya
Source: Antibiotics (Basel). 2021 Jun 24;10(7):772. doi: 10.3390/antibiotics10070772 (PMC8300721; doi:10.3390/antibiotics10070772)
Supplement: Supplementary file 1 [file antibiotics-10-00772-s001.zip › antibiotics-1205485-supplementary.pdf]

antibiotics

Supplementary Table

Table S1. Antimicrobial resistance profile of staphylococci recovered from dairy cows' mastitis cases from two counties, Kenya.

Isolate ID	Antibiotic Resistant gene expressed	Phenotypic resistance profile
457(1)[1]	*blaZ, tetM, ermB, msrA*	AMP, FOX, ERY, TET
803(1)[2]	*blaZ, strB,msrA*	AMP, FOX, STR, ERY, NX
657[1]	*blaZ, tetM, ermB*	AMP, FOX, TET
379[1]	*blaZ,strB,msrA*	AMP, SXT, STR, ERY, TET
525[1]	*blaZ,strB,msrA*	AMP, STR, ERY, TET
1510(1)[2]	*blaZ,tetM,msrA*	AMP, FOX, C, ERY, TET, CIP
244[2]	*tetM,strB,ermB*	AMP, FOX, SXT, STR, GEN, TET, C
1530[2]	*blaZ, strB, ermB*	AMP, FOX, SXT, STR, GEN, ERY, TET, CIP, C
683[2]	*blaZ,tetM,strB*	AMP, FOX, STR, TET
37[1]	*blaZ,strB*	AMP, FOX, SXT, STR, ERY, TET
65(4)[1]	*blaZ, msrA*	AMP, FOX, SXT, STR, ERY, GEN, TET, C
242(2)[1]	*blaZ, strB*	AMP, FOX, STR, ERY, GEN, TET, C
247(2)[1]	*blaZ, ermB*	AMP, FOX, STR, GEN, ERY, TET, NX, C,
5(KRF)[1]	*blaZ, msrA*	AMP, FOX, SXT, ERY
482(2)[1]	*blaZ, tetM*	AMP, FOX, TET,
1338[1]	*blaZ,msrA*	AMP, SXT, ERY, CIP
908(1)[1]	*blaZ,tetM*	AMP, STR, SXT, TET
253(1)[1]	*blaZ,tetM*	AMP, TET
187[1]	*blaZ, strB*	AMP, STR, TET
474[1]	*blaZ,strB*	AMP, SXT, STR, ERY, TET
806[2]	*blaZ, strB*	AMP, TET
1512[2]	*blaZ, tetM*	AMP, TET
1604[2]	*blaZ, ermB*	AMP, FOX, SXT, ERY, TET
1579[2]	*blaZ, msrA*	AMP, SXT, STR, GEN, ERY, C
1247[2]	*blaZ, tetM*	AMP, TET, C
1189[2]	*blaZ, strB*	AMP, STR, NX
1609[2]	*blaZ, ermB*	AMP, ERY
472(2)[2]	*blaZ, strB*	AMP, FOX, STR
1178[2]	*blaZ,tetM*	AMP, ERY, CIP, TET

Only staphylococci isolates carrying ≥ 2 antibiotic resistant genes are shown in Table S1; [1]*Staphylococcus aureus*, [2]Coagulase-negative staphylococci, FOX cefoxitin, AMP ampicillin, ERY erythromycin, TET tetracycline, C chloramphenicol, GEN gentamicin, STR streptomycin, SXT trimethoprim-sulfamethoxazole, CIP ciprofloxacin and NX, norfloxacin.
